# A critical assessment of oral care protocols for patients under radiation therapy in the regional University Hospital Network of Madrid (Spain)

**DOI:** 10.4317/jced.52557

**Published:** 2015-12-01

**Authors:** Isabel Lanzós, David Herrera, Eduardo Lanzós, Mariano Sanz

**Affiliations:** 1 ETEP (Etiology and Therapy of Periodontal Diseases) Research Group, University Complutense, Madrid, Spain; 2 Oncological Radiotherapy Service Hospital 12 de Octubre, Madrid, Spain

## Abstract

**Background:**

This research was aimed to critically evaluate, under the light of the available scientific evidence, the oral care protocols recommended by different hospitals in head and neck cancer (HNC) patients under radiation therapy.

**Material and Methods:**

A questionnaire requesting all the relevant information for the oral care of these patients was sent to the 9 University Hospitals in Madrid. The answers were categorized and analyzed. In addition, an electronic search was conducted to identify the most relevant papers (systematic reviews [SR] and randomized clinical trials [RCTs]) assessing oral care protocols for patients treated for HNC with radiation therapy.

**Results:**

Eight out of nine centers answered the questionnaire and the retrieved information was tabulated and compared. These recommendations were analyzed by a computerized search on MEDLINE and the Cochrane Oral Health Collaboration Database. The results of the analysis clearly shown a great heterogeneity, in terms of oral health care protocols, regarding the management of irradiated patients (for HNC) within the Hospitals of Madrid region. In addition, some of the recommendations lack solid scientific support.

**Conclusions:**

The present survey revealed that the recommendations provided by the different hospitals were clearly different. The available evidence, supported by SR and RCTs, suggested the need of an oral assessment before cancer treatment, in order to prevent and treat dental pathologies and avoiding potential complications; during cancer treatment, it is relevant monitoring the patient in order to decrease the severity of the side effects, and to avoid any tooth extraction or surgery and special attention should be paid to mucositis, xerostomia and candidiasis; after cancer treatment, the following are relevant aspects: the risk of osteoradionecrosis, trismus, caries and the risks associated to dental implants.

** Key words:**Head and neck cancer, supportive care in cancer, radiotherapy complications, management and oral care on cancer treatment.

## Introduction

The treatment of patients suffering from head and neck cancer (HNC) is increasingly more effective. The National Cancer Data Base reflects an increase in the overall survival from 45.5% in 1994 to 53.4% in 2005, with concurrent chemo-radiotherapy (1). These therapies, however, are frequently associated with side effects, both in short- and long-term. Among these side effects, oral pathologies are frequent and a source of impairment of the patient’s health and wellbeing (2). Even though there are effective preventive and therapeutic agents to manage these oral complications, there are no standard protocols with evidence based efficacy and the collaboration between the different medical specialists treating these patients and oral health personnel when these patients are hospitalized is usually deficient (3). It is therefore the aim of this descriptive study to assess the existing oral care protocols for the treatment of these patients in a public health hospital network, to analyze their rationale and scientific base and to ultimately provide recommendations based on evidence-based efficacy.

## Material and Methods

Two approaches to identify protocols of oral care for irradiated HCN patients were performed, namely a questionnaire for Hospital and a literature review.

-Questionnaire: The Radiotherapy units of the Oncology Services of the University Public Hospital Network in the Region of Madrid (Spain) were contacted (by mail) requesting their protocols in oral care for the treatment of the HNC patients, before, during and after the application of radiotherapy. The received information was properly tabulated and categorized into the different components of the specific protocol.

-Literature review: The evidence base of these recommendations was analyzed by a computerized search on MEDLINE and the Cochrane Oral Health Collaboration Database, analyzing the efficacy of the different specific therapies with special focus on randomized clinical trials (RCTs) and systematic reviews (SRs) that could help in the support the proposed recommendations.

## Results

▪ Results: questionnaire

Eight of the nine (88.8%) University Hospitals in the Region of Madrid answered the request (Ramón y Cajal, Puerta de Hierro, Clínico San Carlos, 12 de Octubre, Gregorio Marañón, La Princesa, La Paz, Hospital de Madrid Clara Campal). In  Table 1, different oral care therapeutic recommendations provided by each hospital are listed, being the most frequent advices: assessment by dentist, use of alcohol-free antiseptics, adequate hygiene oral (soft toothbrush and fluoride toothpaste), nutritional recommendations, and to avoid drinking and smoking. Regarding the use of rinses, some centers recommended a particular active agent, while others do not specify the agent or the frequency of use.

**Table 1:**
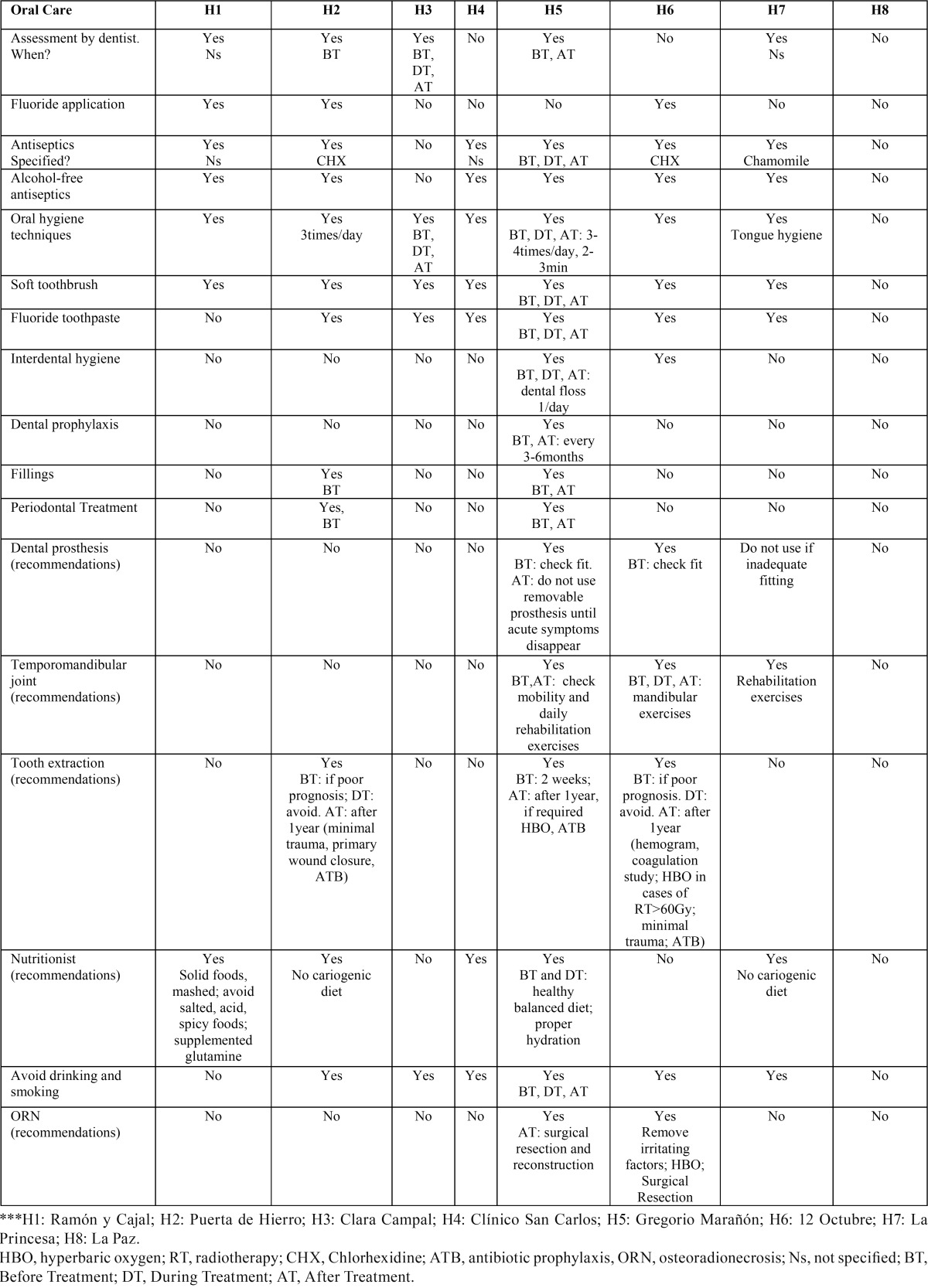
Recommendations, for oral care, as administered by the different surveyed centers.

 Table 2 shows the recommendations for the treatment of mucositis, with seven centers (87.0%) recommending a mixed solution with chamomile and/or bicarbonate. Five centers (62.5%) recommended, in case of pain, the use of analgesics and an-ti-inflammatory, two centers (25%) recommended topical anesthetics. Four centers (50%) recommended nutritional advice through a nutritionist. One center (12.5%) recommended coating agents of the mucosa. In case of candidiasis only one hospital (12,5%) recommended treatment.

**Table 2:**
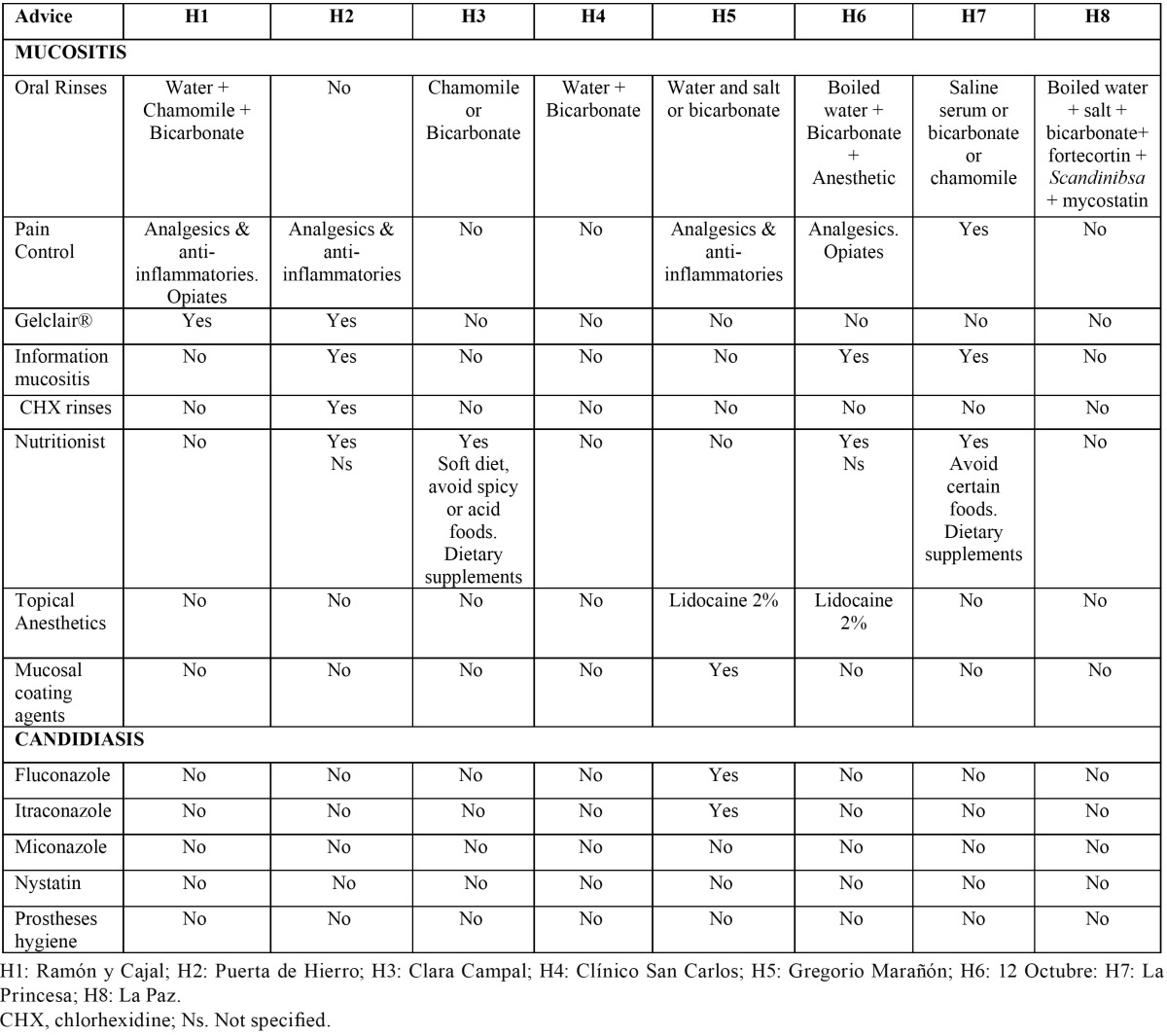
Recommendations, in case of mucositis and candidiasis, as administered by the different surveyed centers.

 Table 3 lists the recommendations in case of xerostomia. Five hospitals (62.5%) included a general definition of this condition and reported general measures as the increase of water intake and changes in the diet. In four hospitals (50%) different rinsing solutions were recommended. In three hospitals (37.5%) the use of chewing gum, ice cubes, moisturizing lip products and saliva substitutes were recommended. In one hospital (12.5%), the salivary function was analyzed.

**Table 3:**
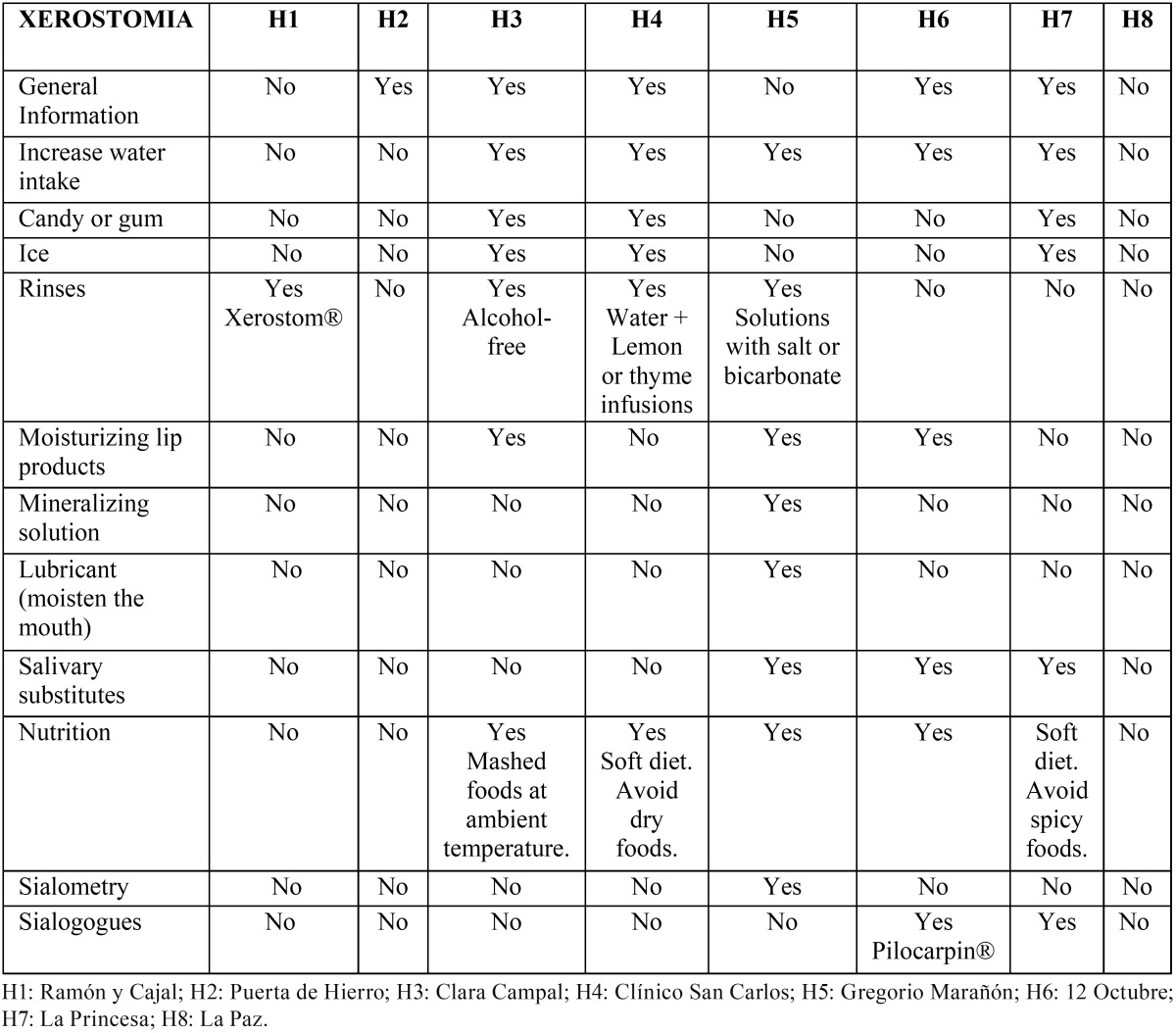
Recommendations, in cases of xerostomia, as administered by the different surveyed centers.

▪ Results: literature review

• Before cancer treatment

- It is highly recommended to perform an oral examination and carry out the proper treatments (if necessary) before the cancer treatment starts (4,5). In this way, the oral problems associated with radiotherapy can be prevented or minimized through the appropriate management, as suggested by the consensus of the National Institute of Health (1989) (4,6). Clinical and radiographic examination is crucial in order to determine the presence of periapical pathology and to evaluate the periodontal and oral health status. For this purpose, both panoramic and selected periapical radiographs should be carried out before radiotherapy treatment (RT).

- It seems essential to have a consultation with the patient’s physician to find out more in order to gain a better grasp of the RT (external beam or radioactive implant), RT characteristics (location, size of the treatment field, RT fractionation, total dose), that are fundamental for the overall risk assessment and scheduling any dental intervention required (3).

- Oral hygiene protocols may decrease the incidence, severity and duration of the oral complications. A soft toothbrush and a fluoride toothpaste or gel should be recommended to prevent the accumulation of plaque and tooth demineralization (3). The use of plaque revealers may be useful in order to help the patient in identifying problematic areas. The proper brushing technique must be tailored for each patient, as well as the description of wrong habits and the importance of the control of interdental plaque, either with floss or interdental brushes (7).

- The use of products containing fluoride reduces the risk of caries in patients under RT treatment (4,5). To prevent “rampant” tooth decay, custom trays with neutral sodium fluoride gel (1.1%) should be applied for 5 min on a daily basis, beginning the first day of RT and continuing on a daily basis while dry mouth and saliva flow remains low (3).

- Supragingival prophylaxis or subgingival scaling (in patients with periodontitis) (4).

- Treatment of carious or endodontic lesions before cancer treatment (4).

- It is convenient to perform a sialometry to check the salivary flow rate (both resting and stimulated) since it can be altered during treatment.

- Adjustment or modification of removable prosthetics that do not properly fit, in order to avoid erosions or mucosal lesions.

- Tooth extractions: the criteria for tooth extractions are not universally accepted and should be subjected to clinical judgment (4). Tooth extraction must be non-traumatic and obtaining primary closure, allow ten days between extraction date and granulocyte count <500/mm3, avoid intra-alveolar hemostatic packing agents, and platelet transfusion if platelet count <40,000/mm3, or prophylactic antibiotics if granulocyte count <2,000/mm3 (4).

• During cancer treatment

Monitoring the patients during cancer treatment must be increased in order to decrease the severity of the side effects. It is necessary to instruct the patient to avoid any tooth extraction or oral surgery during cancer treatment.

During cancer treatment, when the mechanical control of the plaque becomes more difficult, the use of a chemical plaque control with chlorhexidine (CHX) mouth rinses can be beneficial (8,9) to prevent the occurrence of microbial infections, gingival inflammation, bleeding and to reduce the risk of caries (4,10). Mouth rinses with 0.12% CHX have antibacterial and antifungal properties, resulting in an effective anti-plaque and anti-gingivitis effects. It should be used in formulations with adequate bioavailability and without alcohol to avoid tissue irritation (3,10).

* During cancer treatment: mucositis

- The patient must be informed about the possibility that mucositis can occur and the available measures to alleviate it.

- Maintaining proper oral hygiene can reduce its incidence (3,11).

- Avoid smoking and drinking alcohol.

- Remove the prostheses during the acute phase of mucositis, in order to avoid further complications.

- Nutritional support and appropriate hydration (5): small and frequent meals are recommended, favoring foods rich in proteins and calories. The use of foods having soft, pasty or semi-liquid consistency, easy to chew and swallow, with mild flavors at room temperature, are suggested; excluding those spicy, salty, and acidic. It is also helpful to promote liquid intake (2-3 liters/day) (12).

- Pain control: pain can be relieved with the use of topical or systemic anesthetics. In case of mucositis with mild to moderate pain, topical anesthetics such as benzocaine or viscous lidocaine can be used. Topical steroids (diphenhydramine or dexamethasone) are sometimes used to reduce the inflammatory reaction of the oral mucosa (6). Rinses with 0.5% doxepin may be effective in treating pain from oral mucositis (11,13). In the most severe cases, rinses with 2% morphine can keep pain under control (11,13,14).

- Treatment with low-intensity laser reduces the severity of mucositis, as it relieves or cures ulcers (13-16).

- Interventions such as cryotherapy (“chips” of ice) and Keratinocyte Growth Factor (Palifermin®, Swedish Orphan Biovitrum. Stockholm, Sweden) have shown evidences of being beneficial in preventing mucositis in patients irradiated for HNC (2,13). There is strong evidence in favor of 0.5% benzydamine hydrochloride mouth rinses for the prevention of oral mucositis in patients receiving moderate doses of RT for HNC (up to 50 Gy) with or without chemotherapy (CT) (6,13).

* During cancer treatment: xerostomia

Patients with xerostomia should increase fluid intake to 25-30 ml/kg/day, preferably with acid juices to stimulate saliva production; the meals should be moist, soft, with sauces, in the shape of puree, broths and ice creams; a humidifier should be used at night; avoiding smoking and drinking alcohol (12). The intensity-modulated radiotherapy (IMRT) can reduce the adverse effect of xerostomia (5,17). There is no strong evidence for a specific topical treatment being effective for treating the symptoms of dry mouth (18). In patients with residual salivary function, saliva stimulants (sugarless gum, pilocarpine and cevamaline, that increase the salivary fluid in patients with radio-induced xerostomia) may be useful (5). Patients without salivary function can get benefits from the use of artificial saliva or other fluids to keep the mucosa hydrated.

* During cancer treatment: candidiasis

For the prevention of oral candidiasis, there is strong evidence that suggests that those drugs totally (fluconazole, ketoconazole, itraconazole) or partially (miconazole, clotrimazole) absorbed by the gastrointestinal tract, can prevent candidiasis in patients irradiated for HNC. There is also evidence that these drugs are more effective than drugs not absorbed by the gastrointestinal tract (19,20).

• After cancer treatment

A program of post-RT visits shall be scheduled, on a tailored basis, to manage chronic complications and to solve any oral condition that had been postponed (4).

* After cancer treatment: tooth extraction and osteoradionecrosis

It has been known for a long time that ionizing irradiation, among other factors, can delay skin and bone wound healing and that the healing process is closely related to the radiation doses. After tooth extraction in irradiated HNC patients, the bone healing could be delayed or impaired (21). The impact of osteoradionecrosis (ORN) is lower in patients irradiated by IMRT. It is more frequent in the mandible than in the maxilla. This process can occur spontaneously or it can be caused by trauma (e. g. tooth extraction) or oral infections (4,22). The hyperbaric oxygen (HBO) is considered as an adjunctive therapy for ORN, normally in combination with surgery, and it has been associated with better success rates than surgery alone (3) and reduce the risk of ORN following tooth extraction in the irradiated area (23).

* After cancer treatment: caries, Plaque (PI) and Gingival (GI) indices

Patients after antineoplastic therapy had higher PI and GI than healthy patients. The prevalence of dental caries in post-RT and postchemo-RT patients was 24% and 21.4%, respectively (4). The use of fluoride products reduces caries activity in post-RT patients. The use of CHX rinses reduces plaque scores and oral mutans streptococci scores. With dental restorations, conventional glass ionomer cements performed poorer than the comparative materials, specifically amalgam, resin-modified glass ionomer and composite resin restorations (4).

* After cancer treatment: trismus

The prevalence is lower in patients receiving IMRT. There are no clear guidelines for trismus prevention and/or treatment (24). The treatment is physical, with motion exercises to maintain and relax, manual stretching and joint distraction. The trismus in these patients usually has refractory nature and treatment with electrotherapy and pentoxifylline can improve the established trismus (25).

* After cancer treatment: rehabilitation with dental implants

There is consistent scientific evidence to demonstrate that there is a higher failure rate in patients previously treated with RT (26,27). This should be interpreted with caution, due to the difficulties in achieving reproducible experiments and the very limited literature available. According to a narrative review (28), different factors must be considered: RT type, dose, fractionation, use of chemotherapy, risk of recurrence, anatomical region for implant placement, time from RT to implant placement [many researchers have recommended to wait at least 12 months after RT (29) although other researchers have recommended at least 2 years]. Other factors that may be relevant including the time between the 1st and 2nd phase (osseointegration in the irradiated tissue may be slower, so the time between phases should be extended to 4-8 months), prosthetic retention system used, loading factors, management of soft tissues, and the risk of ORN. HBO therapy in irradiated patients requiring dental implants may not offer any appreciable clinical benefits. Additional RCTs are necessary in order to ascertain the effectiveness of HBO in irradiated patients who require dental implants (28).

## Discussion

According to these results, it was necessary to carry out a critical evaluation of the recommendations, in order to evaluate them under the light of the scientific evidence and, after a suitable analysis, to select those properly validated. Therefore, a number of recommendations could be given, based on the best available evidence ( Table 4). It is evident that almost all centers advise patients to have a thorough dental exam before starting RT treatment in order to diagnose and treat possible existing pathologies. In addition, patients are advised to carry out a proper oral hygiene, nutrition and hydration, and also to avoid alcohol use and smoking. Nevertheless, only few centers emphasize how a proper oral hygiene must be carried out. Many centers specify the use of soft brush together with fluoride toothpaste, however, very few point out the need of interdental and tongue hygiene, and none of them specify which is the suitable technique for oral hygiene. In regards to mouth rinsing, many centers advise the patients to carry out rinses. Nevertheless, only few of them specify the rinse type and the frequency of use. Only few centers advise the daily use of fluoride in custom trays to help to prevent dental decay, and the need to perform the proper dental treatments (prophylaxis, dental fillings, fit of dental prosthesis, etc.) in case they were necessary; as well as, the need of assessing, quantitatively, the salivary flow. Ultimately, the decision on the type of oral-dental treatment is based on the clinical and radiographic assessment (including pulp and periodontal problems) of the involved tooth, the available time before cancer treatment and the immune status of the patient, as well as the planned radiotherapy treatment (doses, area to be irradiated, etc.) (6). In those cases in which dental extractions were necessary, only three centers point out the possible patterns to follow. However, within the information related to mentioned patterns, the healing period after tooth extraction and prior to irradiation is not clearly enough pointed out in all of them. A healing period between 10-14 days should be granted, since a delayed bone healing after tooth extraction in irradiated HNC patients has been demonstrated (30).

**Table 4:**
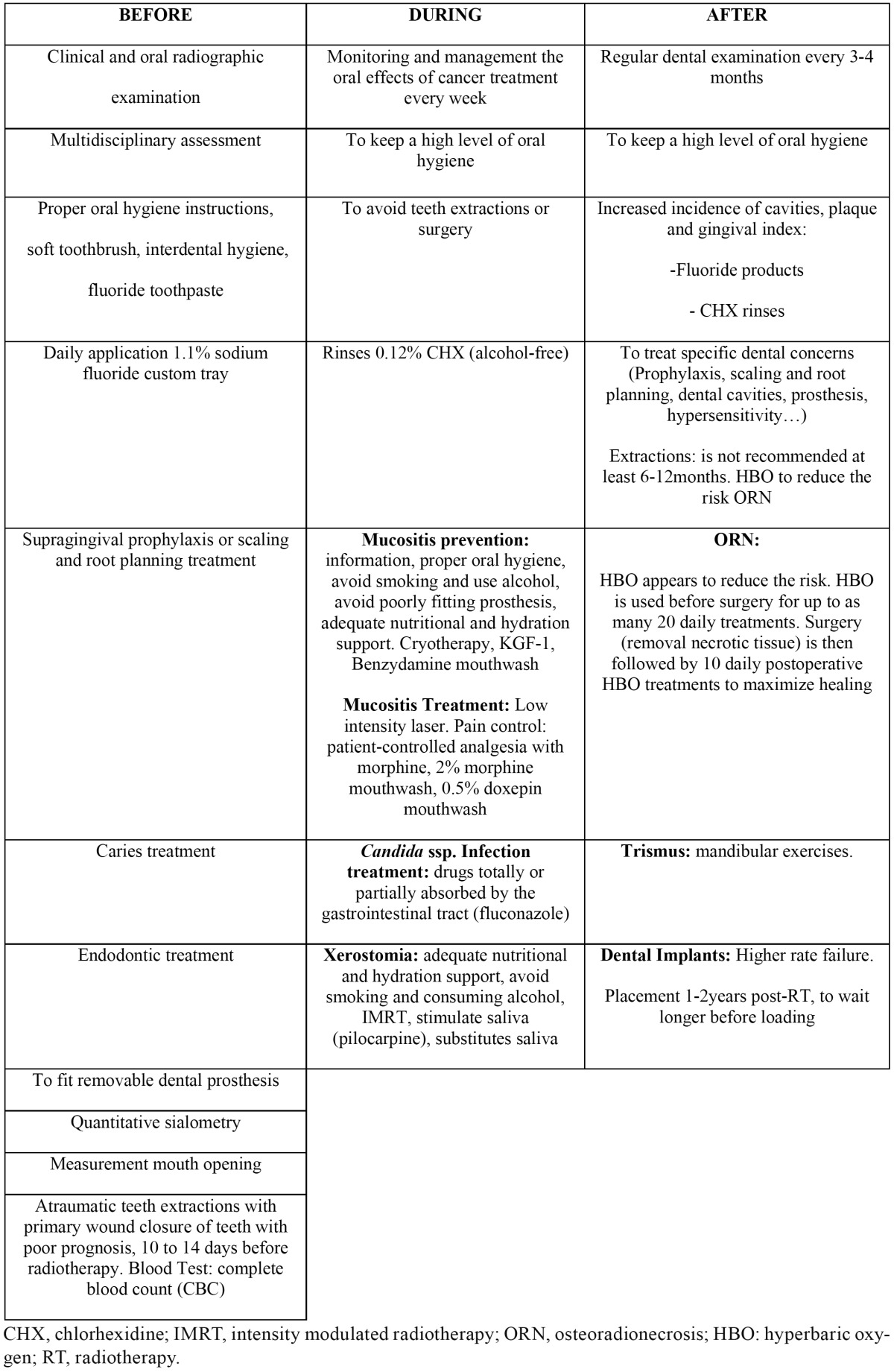
Evidence-based oral care protocol for irradiated patients for head and neck cancer.

The need of dental monitoring during the oncologic treatment is pointed out by very few centers. It is, however, essential to carry it out in order to reduce and/or control the complications that may arise. As it can be seen when reviewing the IMRT literature, it is one of the measures that may contribute to reduce the incidence of complications that may arise, and to avoid any tooth extraction or oral surgery during cancer treatment.

When the measures implemented by the different centers in case of mucositis were analysed, it was noted that many of them recommend that mouth washing must be carried out. Many active ingredients, or the combination of some of them, have been suggested for clinical use, but few RCTs have been carried out (4,5). Therefore, these products must be used with caution since the combination of products from different mouth rinses can interact among them; in addition, excessive dilution can make them ineffective. The review of the literature reflects that, at present, the most effective measures in order to prevent mucositis are cryotherapy, keratinocyte growth factor and mouth rinsing with benzydamine hydrochloride. When mucositis is already established, the most recommendable way of treatment is the low-intensity laser therapy. Little evidence-based literature exists on management strategies for oral mucositis pain. Topical anesthetics, such as viscous lidocaine, are used for mild to moderate pain. Systemic analgesics have been the cornerstone of pain management for patients with oral mucositis experiencing moderate to severe oral pain. The World Health Organization recommends morphine sulfate as the opioid of choice. As to the candidiasis treatment, these infections usually respond well to antifungal but only one center points out the use it. Most of the hospitals inform the patient that xerostomia may arise, and emphasize in regards to keeping the suitable diet, hydration and oral care. However, very few centers recommend the application of ice and chewing sugarless gum or the use of saliva stimulants or substitutes.

As it can be seen in the tables, very few hospitals point out the need of post-RT dental check-ups. Due to a higher incidence of decay, and increases in PI and GI (4), the importance of the check-ups should be stressed. Patients must be advised about the use of fluoridated products and to carry out rinses with CHX. Only few hospitals point out the possibility that ORN can occur and how to solve it. The scientific evidence indicates that IMRT and HBO may contribute to reduce the risk. In case of trismus, only three centers refer to this type of pathology. The dentist should measure in millimeters the distance that patients can open their mouth at baseline and periodically during the follow-up period, and guided exercises may help to prevent it. No hospital refers to the dental implant treatment, but RT induces changes in hard and soft tissues and these changes are harmful to implant survival.

## Conclusions

Heterogeneous and non-solidly scientifically based oral care recommendations are given by Hospitals in Madrid for irradiated HNC patients. According to the available literature, an evidence-based protocol should include actions before, during and after cancer treatment: before, a multidisciplinary assessment and carrying out appropriate dental treatments to avoid complications; during, avoidance of tooth extraction or oral surgery, use of intensity-modulated radiotherapy, instructions to avoid smoking and alcohol use, and nutritional support and appropriate hydration; and after therapy, management of chronic complications and any relevant oral condition should be accomplished, taking into account the risk of osteoradionecrosis.
